# Identification of Priority Areas for Soil and Water Conservation Planning Based on Multi-Criteria Decision Analysis Using Choquet Integral

**DOI:** 10.3390/ijerph17041331

**Published:** 2020-02-19

**Authors:** Haibo Zhang, Jianjun Zhang, Shouhong Zhang, Chunxue Yu, Ruoxiu Sun, Dandan Wang, Chunzhu Zhu, Jianan Zhang

**Affiliations:** 1 School of Soil and Water Conservation, Beijing Forestry University, Beijing 100083, China; zhanghbmail@163.com (H.Z.); zhangs@bjfu.edu.cn (S.Z.); sunruoxiu@bjfu.edu.cn (R.S.); www.wangdandan.net@163.com (D.W.);; 2 Jixian National Station for Forest Ecosystem Research, Linfen 042200, China; 3 Research Center for Eco-Environmental Engineering, Dongguan University of Technology, Dongguan 523808, China; yucx@dgut.edu.cn; 4 School of Information Science & Technology, Beijing Forestry University, Beijing 100083, China; zhuchunzhu@catt.cn

**Keywords:** soil erosion risk, multi-criteria decision analysis, Choquet integral, Loess Plateau

## Abstract

Soil erosion risk assessment is an essential foundation for the planning and implementation of soil and water conservation projects. The commonality among existing studies is that they considered different indicators (e.g., rainfall and slope) in order to determine the soil erosion risk; however, the majority of studies in China neglect one important indicator, namely the slope aspect. It is widely accepted that the vegetation and distribution of rainfall differs according to the different slope aspects (such as sunny slope and shady slope) and these attributes will accordingly influence the soil erosion. Thus, existing studies neglecting this indicator cannot reflect the soil erosion well. To address this problem, a flexible soil erosion risk assessment method that supports decision makers in identifying priority areas in soil and water conservation planning was developed in the present study. Firstly, in order to verify the impact of the slope aspect on soil erosion, field investigations were conducted, and its impact on the characteristics of the community in the study area was analyzed. Secondly, six assessment indicators were selected, including slope gradient, precipitation, NDVI, land use, soil texture and slope aspect. Next, a developed multi-criteria decision analysis (MCDA) method based on the Choquet integral was adopted to assess the soil erosion risk. The MCDA method, combining objective data with subjective assessment based on Choquet integral, could solve the weight problem encountered when using the quantitative method. The parameters required can be modified according to the soil erosion types, assessment scales, and data availability. The synergistic and inhibitory effects among the soil erosion parameters were also considered in the assessment. Finally, the soil erosion risk results in the Xinshui River watershed revealed that more attention should be paid to the slope of farmland and grassland during the planning and management of soil and water conservation projects. The methodology used in the current study can support decision makers in planning and implementing soil and water conservation measures in regions with different erosion types.

## 1. Introduction

Soil erosion is one of the most notable environmental problems worldwide. It has resulted in many challenges for humans, such as the loss of arable land [1], the increase of landslides and debris flows [2], the rise of downstream riverbeds [3,4], and so on. In order to address this problem, priority areas usually need to be identified for the implementation of soil and water conservation based on soil erosion risk assessment. Thus, it is important to develop effective methods for soil erosion risk assessment.

According to existing studies, many methods have been described for soil erosion risk assessment [5,6,7]. The most commonly used assessment methods can be divided into two categories. The first category involves the use of quantitative methods (such as soil erosion models) that are based on sophisticated modeling of the soil loss amount to characterize the soil erosion risk [8,9,10,11,12,13]. Such quantitative methods are usually designed to estimate certain types of soil erosion, such as the wind erosion model (IWEMS) and the water erosion model (RUSLE), based on complex calculations and large amounts of data [14]. However, multiple types of soil erosion usually occur simultaneously in one area, and thus a single quantitative method is hardly suitable for integrated soil erosion assessment [5,15,16]. Meanwhile, the data requirements might constrain their application in data-poor regions [17,18]. In order to address these problems, a second category of qualitative methods has been proposed to assess the soil erosion risk and identify priority areas with higher erosion risk. These qualitative methods have less data requirements and can synchronously identify the key driving factors and processes of different types of soil erosion [19,20]. For example, a previous study by Vrieling et al. (2006) [18] assessed the regional soil erosion risk using two indicators, namely the vegetation cover and slope, and proved that the accuracy of quantitative methods is acceptable. 

Generally, the commonality between the two categories of assessment methods is that they consider different indicators (e.g., rainfall and slope) to determine the soil erosion risk; however, they both neglect one important indicator, the slope aspect. It is widely accepted that the vegetation and distribution of rainfall differs according to the different slope aspects (such as sunny slope and shady slope), and these attributes will accordingly influence the soil erosion. Thus, existing studies neglecting this indicator cannot reflect the soil erosion well [21]. In order to improve the effectiveness of the soil erosion risk assessment method, the slope aspect was investigated and considered in the present study. Meanwhile, the weights used in qualitative methods are usually determined according to the characteristics of the specific region, which is not universal and conducive to the promotion of research results [22,23,24]. It is, therefore, necessary to adopt a universal method to determine the weights of the indicator in the assessment. 

In response to this requirement, a flexible soil erosion risk assessment method that supports decision makers in identifying priority areas for soil and water conservation planning was developed in the present study. Firstly, in order to verify the impact of the slope aspect on soil erosion, field investigations were conducted, and its impact on the characteristics of the community in the study area was analyzed. Secondly, six assessment indicators were selected, including the slope gradient, precipitation, NDVI, land use, soil texture and slope aspect. Next, a developed multi-criteria decision analysis (MCDA) method based on the Choquet integral was adopted to assess the soil erosion risk. The MCDA method, combining objective data with subjective assessment based on the Choquet integral, could solve the weight problem encountered when using the quantitative method. The parameters required can be modified according to the soil erosion types, assessment scales, and data availability with expertise. The synergistic and inhibitory effects among soil erosion parameters were also considered in the assessment in the present study. Furthermore, in order to prove the effectiveness of method proposed the present study, this method was applied to the Xinshui River watershed located in the Loess Plateau, China.

## 2. Materials and Methods

### 2.1. Study Area

**Figure 1:**
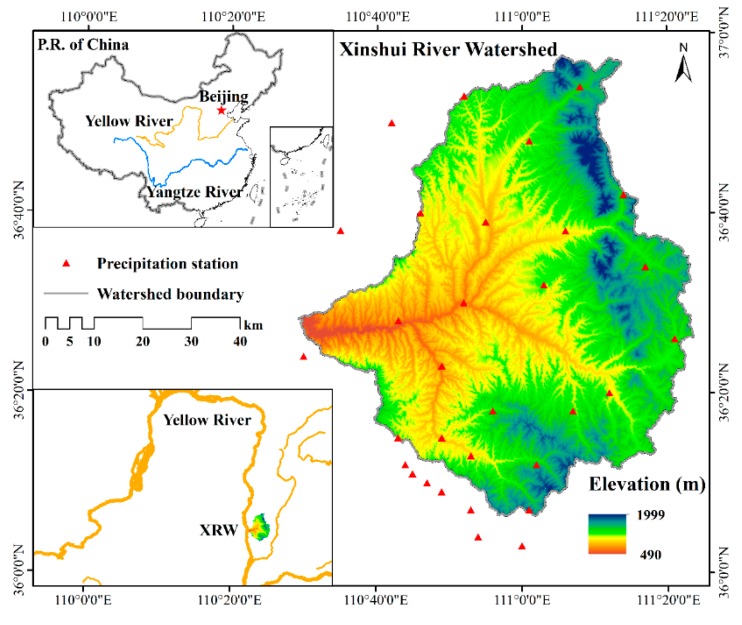
Location of the Xinshui River watershed and spatial distributions of precipitation station.

The Xinshui River watershed (36°6′–36°57′ N, 110°29′–111°23′ E) is situated in the western Shanxi Province, China, and is one of the main tributaries of the Yellow River. The study area is approximately 4326 km^2^. It belongs to the loess hills and gullies region of the Loess Plateau, which is one of the areas with the most serious soil erosion worldwide. More than 60% of the region has soil erosion problems [4], and the average annual soil erosion module during 2000–2010 was reported to be about 1520 t/(km^2^·a) [25]. The main stream has a length of approximately 135 km (Figure 1). This watershed has a semi-arid continental monsoon climate with mean annual precipitation of 515.8 mm, mean annual temperature of 8.5 °C, and mean annual potential evaporation of 1073.7 mm [26]. The altitude in the watershed is between 490 and 2000 m, and the soil type in the watershed is cinnamon soil, which is vulnerable to erosion. At the study area, the primary land use types include forest, farmland, and grassland. The average annual runoff of the watershed is 1.21 × 10^8^ m^3^, the average annual sediment yield is 1.28 × 10^7^ t.

### 2.2. Data Sources

Landsat 5 Thematic Mapper (TM) images from 2015 were used to extract the land use. The spatial distributions of the slope gradients and aspects of the watershed were determined based on the Digital Elevation Model (DEM) data (Table 1). The precipitation data of 29 rainfall stations in or around the watershed (Figure 1) from 2006 to 2015 were transformed into the spatial distribution of the annual average precipitation with a spatial resolution of 1000 m using the Co-Kriging method of ArcGIS 10.1 software (ESRI Inc., California, USA). The NDVI values for 2015 were extracted from MODIS NDVI data (MOD13Q1, NASA EOSDIS Land Processes Distributed Active Archive Center (LP DAAC), South Dakota, USA) [27]. The soil texture data set was acquired from the Chinese Soil Database in the scale of 1:1,000,000.

### 2.3. Field Investigations

The aim of the field investigation conducted in the present study was to explore the relationship between the slope aspect and the characteristics of the community in the study area. The north direction was defined as 0° (flat), shady slope was defined as 0°–67.5° and 337.5°–360°, semi-shady slope was set at 67.5°–112.5° and 292.5°–337.5°, sunny slope was 157.5°–247.5°, and semi-sunny slope was 112.5°–157.5° and 247.5°–292.5°. The field investigations were conducted in the Liugou watershed, which is a part of the Xinshui River watershed. The Liugou watershed covers an area of approximately 1.93 km^2^, while the length of the river watershed is 3 km, and the channel ratio drops is about 0.08. This watershed has been closed for forest cultivation since the 1980s and has been banned for 35 years to date. According to the related regulation of China, the mode of Forest Closure in Liugou watershed is overall closure. During the overall closure period, cutting, grazing, mowing and all other human activities, which do not conducive to the growth and breeding of trees, are forbidden. The forest ecosystem grows naturally. This area was surveyed three times, including in 1996 (banned for 15 years), 2007 (banned for 26 years) and 2016 (banned for 35 years). The survey period was between July and August. A horizontal distance of 10 m was defined as a section. According to the actual conditions of this watershed, a quadrat of about 10 × 10 m or 5 × 5 m in each section was selected to carry out the surveys, and the data collected included the name of plant species, number of individual plants, crown width and so on.

The Jaccard index (*I_J_*). can be used to estimate the similarity degree of the two communities, and the equation is as follows:

*I_J_* = *J*/(*A* + *B* − *J*) (1)

where *J* is the common species of the two quadrats, *A* is the number of species in environmental *Ⅰ*, and *B* is the number of species in environmental Ⅱ. According to Jaccard’s dissimilarity coefficient principle, the similarity degree is defined as follows: *I_J_* = 0 to 0.25 indicates extreme dissimilarity; *I_J_* = 0.25 to 0.50 indicates moderate dissimilarity; *I_J_* = 0.50 to 0.75 indicates moderate similarity; and *I_J_* = 0.75 to 1.00 indicates high similarity.

### 2.4. Soil and Water Conservation Patch (SWCP)

In the present study, the SWCP is defined as a spatial unit with homogeneously geographical and environmental conditions with regards to soil erosion, and is used as the smallest spatial unit for soil erosion risk assessment. This definition was first introduced by Luo (2015) [32] for soil erosion monitoring, evaluation, prevention and other management activities. The criteria for partition SWCP can be modified according to the erosion type, data quality and the minimal area for management.

### 2.5. MCDA Method

In recent years, MCDA has been introduced as a new qualitative approach for the evaluation of the soil erosion risk [7,15,33], and has been applied in various environmental fields due to its flexibility and ability for accommodating a variety of data types (quantitative and qualitative). The applications of this method include regional risk assessment [34,35], land management [36], policy making [37], and identification of forest landscape restoration priorities [38]. Previous studies have also suggested that the MCDA method could be adapted to assess soil erosion risk [5,7,15,16]. 

**Figure 2:**
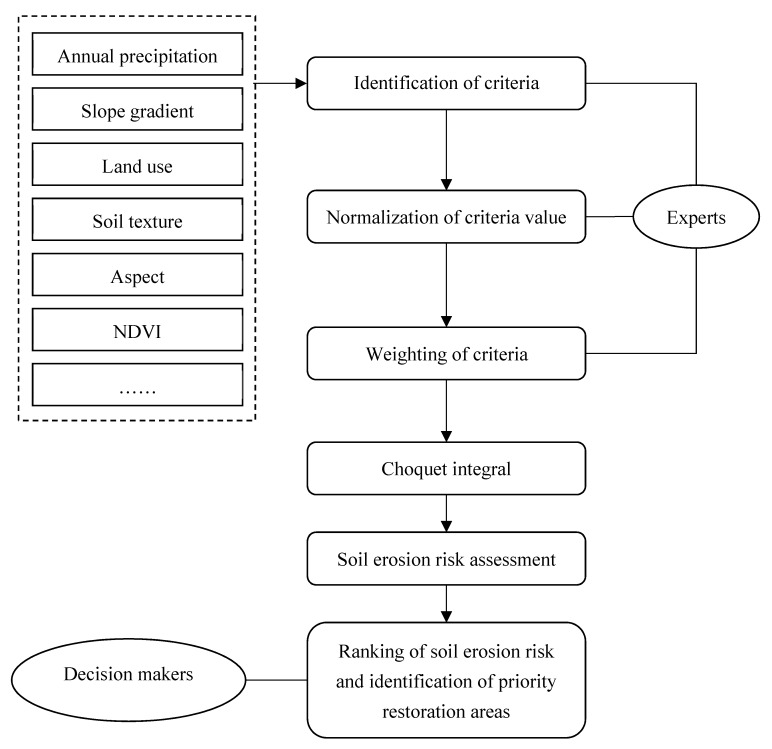
Framework for soil erosion risk assessment.

Thus, the soil erosion risk assessment tool was developed in the present study using the MCDA method, which was proposed by Zabeo, et al. (2011) [39]. The MCDA framework is fundamentally based on the Choquet integral and it allows including expert judgments for synergic and redundant effects between criteria involved in the assessment. Figure 2 shows the MCDA framework for soil erosion risk assessment, which consists of two steps, as follows: 

Step 1: Identification, normalization, and assignment of the weight of relative criteria according to expert judgement;

Step 2: Evaluation and ranking of soil erosion risk, and identification of priority restoration areas based on the outcomes of Step 1.

#### 2.5.1. Identification of Criteria

From the MCDA point of view, each attribute of a SWCP can be considered as a criterion. Suitable criteria for soil erosion risk assessment should be selected according to the soil erosion types and the local conditions, based on the findings of previous studies and expertise. It has been reported that the primary factors controlling soil erosion are the erosivity of eroding agents, soil erodibility, land slope, and vegetation coverage [40]. During the past decade, soil health began to generate widespread interest and considered in the decision making of soil and water conservation. Soil health increases soil erosion decreases and vice versa [41]. Multiple soil and water conservation measures are embodied in soil health. For example, available water holding capacity increases with soil organic matter increase [42], increasing crop surface cover can increase soil aggregation [43] and then prevent surface crusting, increase infiltration, decrease surface runoff, and reduce wind and water erosion [44,45]. However, due to the lacking of soil health indicators data in watershed scale, these kinds of indicators such as soil aggregation, pore structure and aggregate stability were not considered in the study. In the present study, the slope gradient, precipitation, soil texture, land use, slope aspect, and vegetation index (i.e., NDVI) were selected for assessing the soil erosion risk caused by water in the loess hills and gullies region of the Loess Plateau. It is necessary to emphasize that the selection of more attributes for soil erosion risk assessment does not result in better assessment, as too many attributes can increase the difficulty of experts in making a judgment.

#### 2.5.2. Normalization of Criteria Value

**Figure 3:**
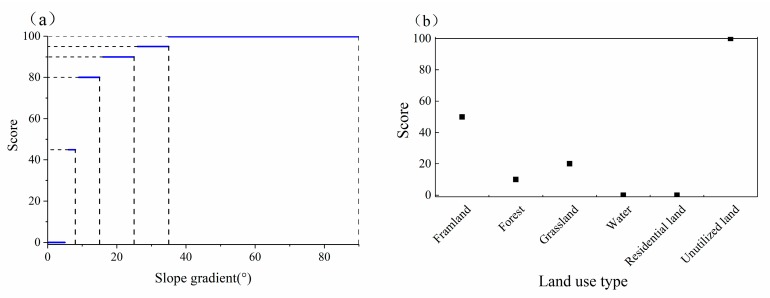
Examples of normalization applied in the case study. (**a**) Numerical variable normalization. (**b**) categorical variables normalization.

The attributes selected for soil erosion risk assessment can be classified into two groups: Numerical variables (such as slope gradient, precipitation, and vegetation index) and categorical variables (such as land use and slope aspect). Since the numerical variables are quantitative and the categorical variables are qualitative, they are not comparable to each other. Hence, a normalization procedure is used to transform all the variables into a “neutral” or “standard” scale. The normalization procedure consists of two steps, as follows: (1) An attribute is classified based on its characters and special principles; and (2) each class is assigned a standard score according to expert judgment. Figure 3 shows examples of the normalization of numerical variables and categorical variables (such as slope gradient and land use, respectively). The numerical variable slope gradient (Figure 3a) is classified into six classes, and each class is assigned a single score to guarantee that the attribute value of slope gradient of each SWCP is homogeneous. Similarly, the categorical variable land use (Figure 3b) is normalized according to the experts’ knowledge and experience. The range of scores assigned to attributes is (0,100), with higher scores representing a greater impact on soil erosion.

The classification and assignment of all the identified attributes for soil erosion risk estimation of the case study discussed in the next sections were provided by experts from the Beijing Forestry University and local policy decision makers. Their judgement was based on previous studies, relative national standards, data distribution features and conventional classification methods. As shown in Table 2, the land use and aspect were classified by the general classification principle and description of land use is shown in Table 3; the slope gradient was classified into 0°–5°, 5°–8°, 8°–15°, 15°–25°, 25°–35°, and >35°, according to the national standard [46]; the precipitation was classified according to the climate conditions for the study area; the NDVI value was classified into five classes with the same intervals; and finally, the soil texture was classified on the basis of the soil texture classification, as defined by the International Society of Soil Science [47]. 

#### 2.5.3. Aggregation of Attribute Values

After normalization, the values of the selected attributes were aggregated into numerical outputs. The aggregation was implemented using the MCDA method based on non-additive measures, which included expert judgment on the synergistic and redundant impact among the selected attributes. Non-additive measures are well known by the terms fuzzy measures, capacities, monotonic measures and monotonic games [48]. The proposed MCDA aggregate function uses Choquet integral to simulate the weighted average behavior in a linear environment, which can be regarded as a nonlinear form of weighted average [39]. The Choquet integral [49,50] is an aggregation operator that is used to generalize the weighted arithmetic mean, and to represent the importance of a criterion and the interactions between criteria [51]. The basics of the Choquet integral and the non-additive measures are explained below:

Given N = {1, 2, 3, … n} the set of the criteria, a non-additive measure is a set function: 

m: S ⊆ N → [0, 1], so that, ∀S, T ⊆ N the following condition holds [52]:

m(∅) = 0, ∀S, T ⊆ N: S ⊆ T ⇒ m(S) ≤ m(T), m(N) = 1

The second condition is a monotonic constraint. A non-additive measure is classified as:

Additive if: m(S ∪ T) = m(S) + m(T), separately effect, Sub-additive if: m(S ∪ T) < m(S) + m(T), redundant effect, Super-additive if: m(S ∪ T) > m(S) + m(T), synergic effect,

where S ∩ T = ∅.

As shown above, a non-additive measure is a monotonic set function that gives each possible subset of the criteria a positive weight, rather than a single criterion. Accordingly, the entire importance of multiple criteria can be less, equal, or greater than the sum of the importance of each individual criterion. Compared with the WA and other similar algorithms that simply compute the scores of alternatives by averaging the values of all possible subsets of criteria, non-additive measures require parameters to be assigned for all the possible combinations of criteria. If there is no interaction between the criteria, then the method will degenerate into a WA algorithm [53].

Questionnaires were designed for experts to define the measure scores associated with each possible combination of the criteria, as shown in Table 4, with each row of the table expressing a possible combination. Value 1 represents the highest class of each criteria values, in other words, the maximum effect in the soil erosion risk evaluation. Value 0 represents the lowest class of each criteria value. Score values are integer numbers in the (0,100) closed set. 

After the criteria measures are defined, the aggregation function can be performed using the Choquet integral [50], which is defined as follows:

Let *μ* be a measure on X, whose elements are denoted as *x*_1_, …, *x*_2_ here. The discrete Choquet integral of a function *f*: X → R+ with respect to *μ* is defined by:

(2) $$C_{\mu }\left(f\right):=\sum_{i=1}^{n}\left(f\left(x_{\left(i\right)}\right)-f\left(x_{\left(i-1\right)}\right)\right)\mu \left(A_{\left(i\right)}\right)$$

where (*i*) indicates that the indices have been permuted so that 0 ≤ *f*[*x*_(1)_] ≤ ⋯ ≤ *f*[*x*_(*n*)_], *A*_(*i*)_: = {*x*_(*i*)_,…, *x*_(*n*)_}, and *f*[*x*_(0)_] = 0.

## 3. Results and Discussion

### 3.1. Relationship between the Slope Aspect and Community Characteristics

**Figure 4:**
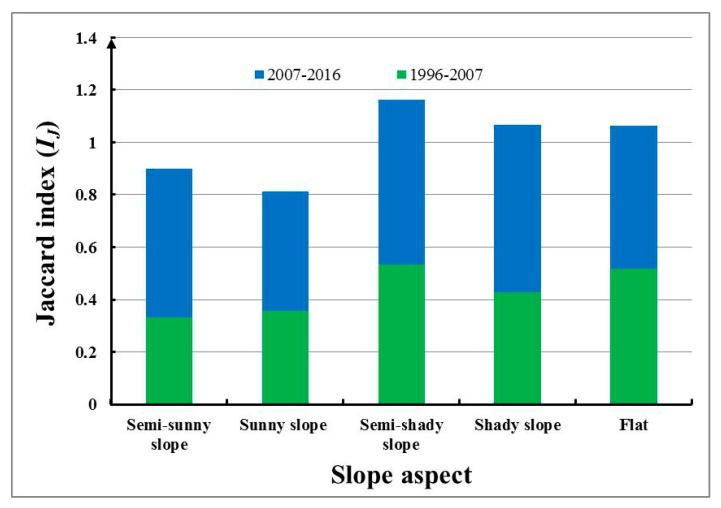
Changes in the *I_J_* index with the time gradient in different habitats.

Figure 4 shows the changes in the *I_J_* index of different habitat communities with the time gradient. Between 2007 and 2016, the Jaccard index of the semi-sunny slope, semi-shady slope, shady slope and flat habitats ranged from 0.50 to 0.75, indicating that the community vegetation types of these four habitats in 2016 were the same as those in 2007. Compared with the community, it is at a moderately similar level without major changes. The index for sunny slope was between 0.35 and 0.45, which indicates a moderately dissimilar level, that is, the water condition is the best and the water condition is the most. The Jaccard index of each habitat in the second period increased significantly compared with that in the first period. Among them, the indices for shady slope and semi-sunny slope increased significantly, with growth rates of 48.5% and 69.6%, respectively. 

### 3.2. Soil Erosion Risk Attributes

#### 3.2.1. Land Use

**Figure 5:**
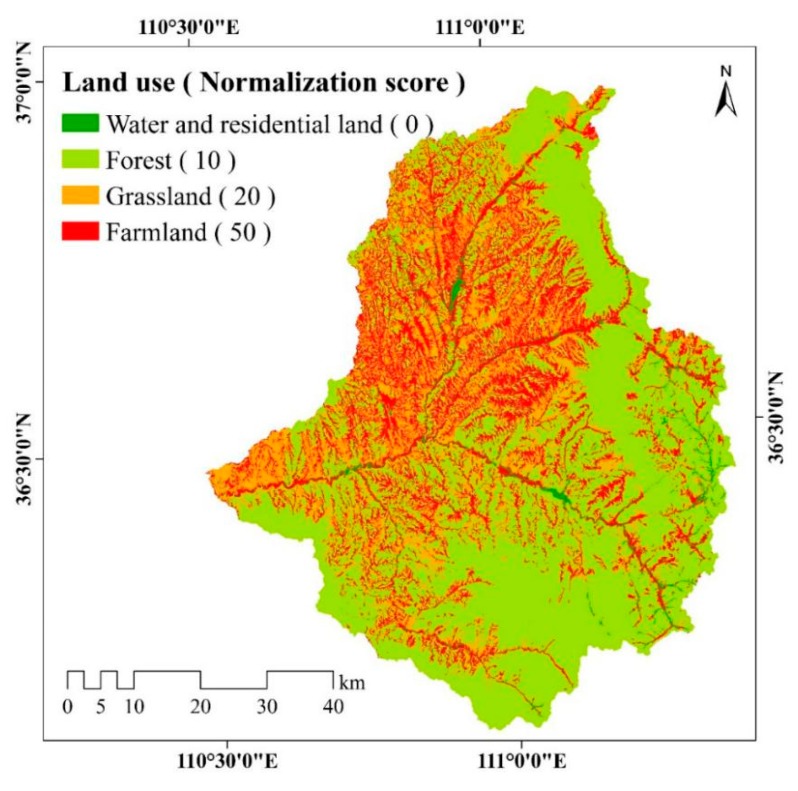
Spatial distribution of Land use in the study area. There are five types of land use including forest, grassland, farmland, water and residential land. Different color represent different types. Higher normalization score of land use represent a greater impact on soil erosion.

The land use and normalization score map shown in Figure 5 illustrates that the largest proportion of the study area is covered by forest land (61.22%), followed by farmland (21.14%), grassland (15.66%), residential land (1.81%) and water (0.18%). Most of the forests are located in the east and south of the watershed.

#### 3.2.2. Slope

**Figure 6:**
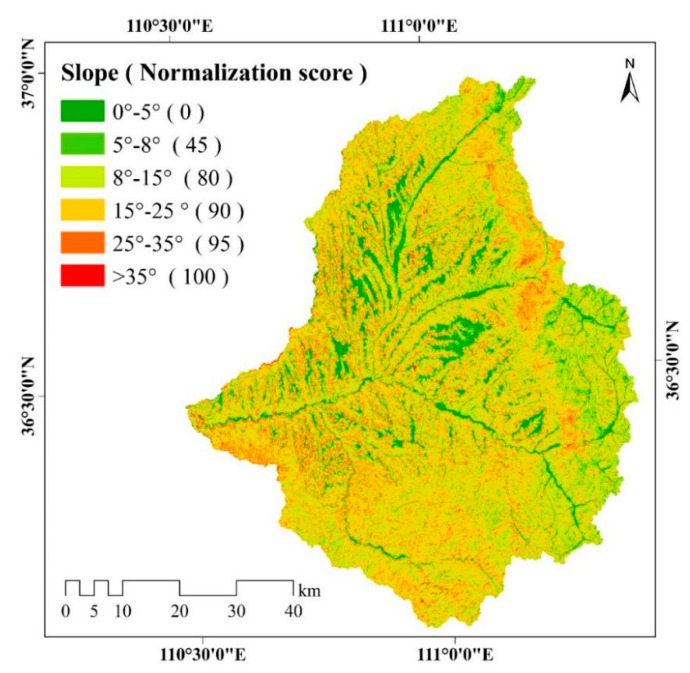
Spatial distribution of slope gradient in the study area. Slope gradient is classified into 0°–5°, 5°–8°, 8°–15°, 15°–25°, 25°–35°, and >35°. The different colors represent different slope classes. A higher normalization score of the slope represent a greater impact on soil erosion.

In the slope gradient map shown in Figure 6, the slope gradient is of Class 3 (8°–15°) and Class 4 (15°–25°) for the largest area of the watershed, accounting for 37.03% and 39.27% of the total area, respectively. Class 1 (0°–5°) slope gradient is mostly distributed near the rivers, which is consistent with the distribution of farmland, and it takes up 10.62% of the total area. Class 2 (5°–8°) and Class 5 (25°–35°) slope gradient cover 5.71% and 7.02% of the total area, respectively, while Class 6 (>35°) has the smallest area ratio (0.35%). 

#### 3.2.3. Slope Aspect

**Figure 7:**
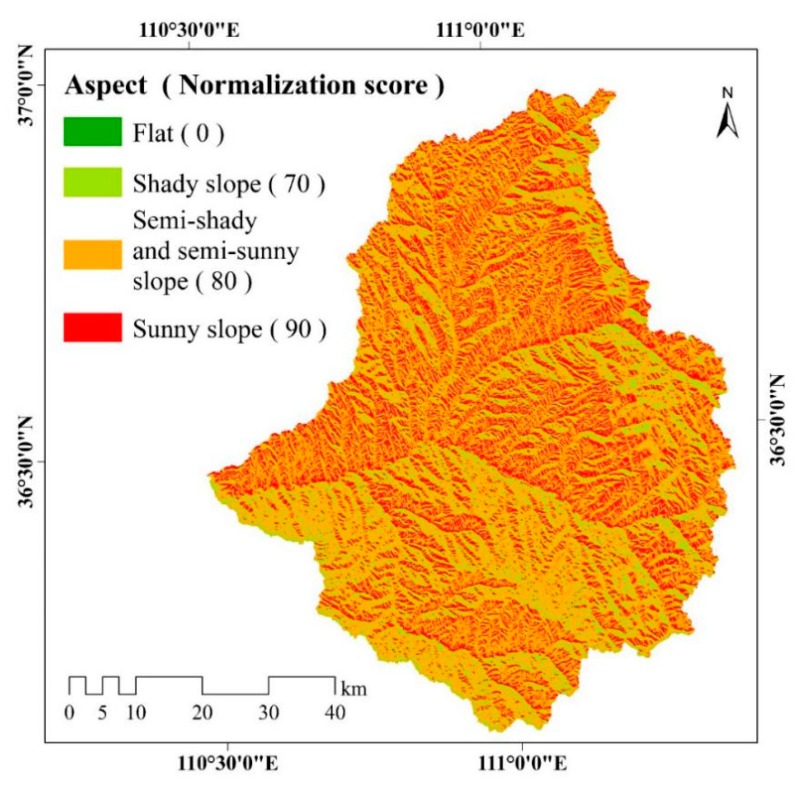
Spatial distribution of slope aspect in the study area. The different colors represent different slope aspects, including flat, shady slope, semi-shady and semi-sunny slope, sunny slope. A higher normalization score of the slope aspect represents a greater impact on soil erosion.

The slope aspect map shown in Figure 7 indicates that most of the aspect of the majority of the (52.29%) corresponds to semi-shady and semi-sunny slopes, while sunny and shady slopes account for 25.11% and 22.60% of the watershed, respectively. Flat area only accounts for 0.003% of the watershed in the study area.

#### 3.2.4. Precipitation

**Figure 8:**
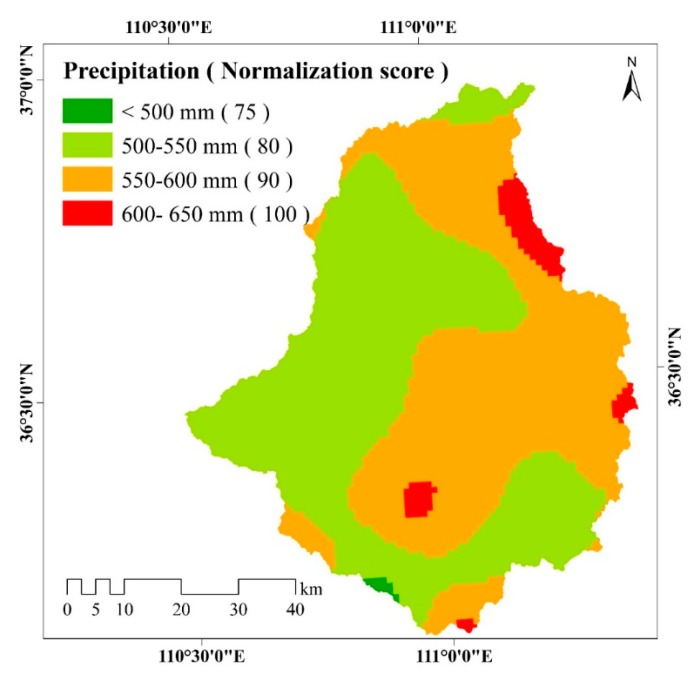
Spatial distribution of precipitation in the study area. Precipitation is classified into four classes: <500 mm, 500–550 mm, 550–600 mm and 600–650 mm. The different colors represent different precipitation classes. Higher normalization score of precipitation represent a greater impact on soil erosion.

Figure 8 shows that the precipitation in the eastern part of the watershed is higher than that in the northwestern area. This is mainly due to the fact that the largest mountain range of the watershed, i.e., the Lüliang Mountain, is located in the eastern part of the study area. Approximately 50.69% of the watershed has an annual mean precipitation value between 500 and 550 mm, and 45.83% has a value between 550 and 600 mm. Only 3.21% and 0.27% of the watershed has an annual mean precipitation value larger than 600 mm and lower than 500 mm, respectively.

#### 3.2.5. Vegetation 

**Figure 9:**
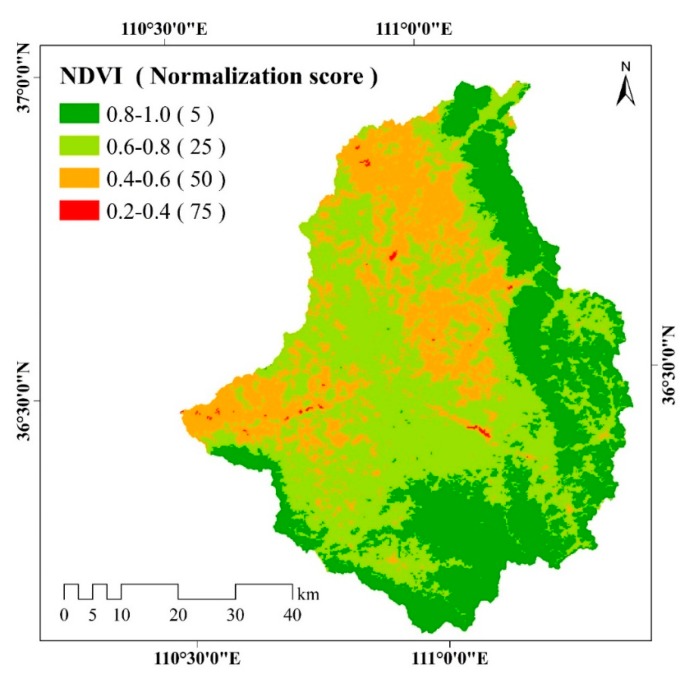
Spatial distribution of NDVI in the study area. NDVI is divided into 4 classes, 0.2–0.4, 0.4–0.6, 0.6–0.8 and 0.8–1.0. Different color represent different NDVI class. Higher normalization score of NDVI represent a greater impact on soil erosion.

The spatial distribution of NDVI shown in Figure 9 indicates that NDVI values among the range of 0.8–1.0 are mostly located in the southern and eastern regions of the study area. The proportions of areas with NDVI values ranging at 0.6–0.8 and 0.8–1.0 are 48.14% and 30.42%, respectively. Areas with lower NDVI values ranging between 0.4 and 0.6, and NDVI values ranging between 0.2 and 0.4 account for 21.26% and 0.18% of the study area, respectively. 

#### 3.2.6. Soil Characteristics

**Figure 10:**
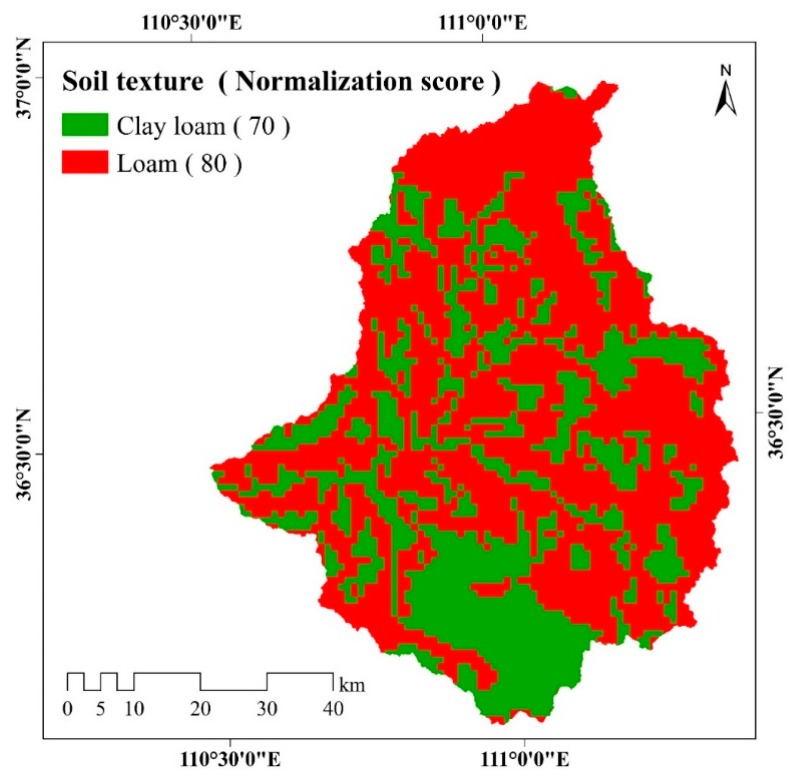
Spatial distribution of soil texture in the study area. There are two types of soil texture including clay loam and loam. The different colors represent the different soil texture types. A higher normalization score of soil texture represents a greater impact on soil erosion.

There are only two soil texture types distributed in the watershed, namely clay loam and loam (Figure 10). Loam is more widely spread than clay loam, and these two soil types take up 62.17% and 37.83% of the watershed, respectively. Clay loam is mainly distributed in the southern region of the watershed.

### 3.3. Distribution of Soil Erosion Risk

**Figure 11:**
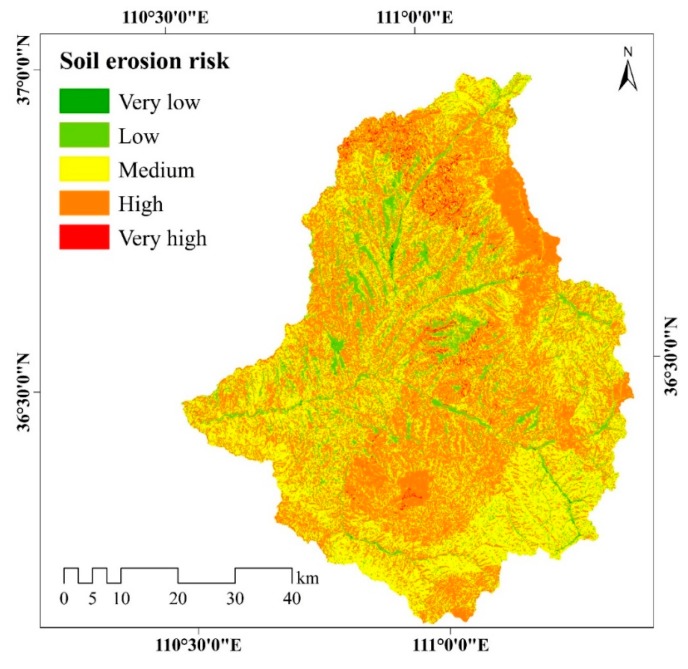
Spatial distribution of soil erosion risk of the Xinshui River watershed. (Very low, score range: ≤5000; low, score range: 5001–6000; medium, score range: 6001–7000; high, score range: 7001–8000; very high, score range: >8000).

Using Equation (2), the soil erosion risk of the Xinshui River watershed was calculated and then grouped into five classes, and the results are presented in Figure 11. Approximately half of the watershed (44.32%) has medium soil erosion risk, with risk scores between 6001 and 7000, while only 0.8% and 0.29% of the watershed was identified to have very high (risk score, >8000) and very low (risk score, ≤5000) soil erosion risk, respectively. A total of 43.13% of the watershed has a high soil erosion risk with scores between 7001 and 8000. These results show that the soil erosion risk is still severe in the Xinshui River watershed, even though the annual soil erosion modulus decreased significantly in recent years. For instance, the average annual soil erosion modulus was 203.09 t/km^2^ between 2006 and 2015. However, this value was 1090.31 t/km^2^ in 2013, and is still larger than the soil loss tolerance (1000 t/km^2^) in the Loess Plateau.

The spatial distribution map of the soil erosion risk indicates that the very high and high erosion risk areas are mostly distributed in the northeastern and southern parts of the watershed, while the low erosion risk areas are distributed in the western and northwestern regions. The high and very high erosion risk is mainly related to the distribution of vegetation and precipitation. In the northern part of the watershed, the NDVI is relatively low and the annual precipitation is high. In addition, the high risk in the northeast and south of the watershed is highly influenced by the high precipitation, even though the vegetation coverage is high. The low erosion risk is mainly distributed in the area with low slope near the river. Moreover, the land use types in these regions are mainly forest land and grassland. Furthermore, the results indicated that the soil and water conservation effect of vegetation is limited when precipitation is high. More than 70% of the soil erosion in the Loess Plateau was caused by rainstorms with high intensities and short duration [54]. For example, according to the observation data in the Daning hydrological station, in 2013, the total sediment yield caused by a continuous heavy rainfall of 194.5 mm and a daily rainfall of 63.5 mm account for 92.04% of annual sediment yield. The annual sediment modulus in 2013 was 1268 t km^−2^ a^−1^, nearly 16 times the value in 2012. 

### 3.4. Priority Areas for Soil and Water Conservation Planning

**Figure 12:**
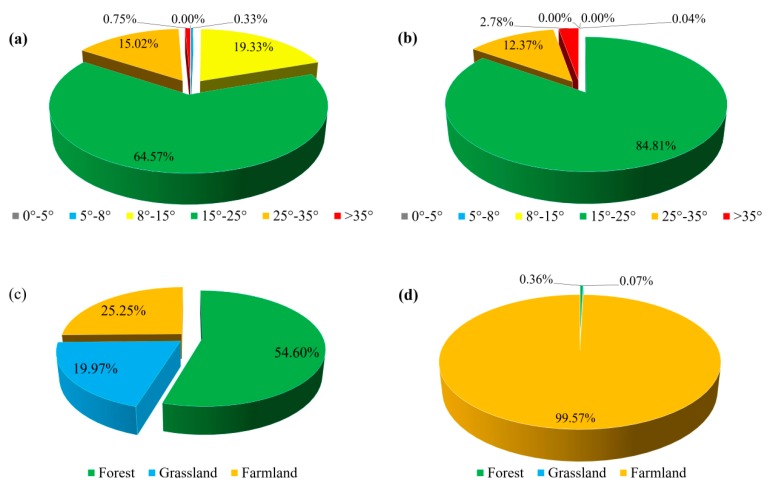
Intersection between high and very high soil erosion risk areas with the slope gradient and land use. (**a**) High soil erosion risk class integrated with slope gradient. (**b**) Very high soil erosion risk class integrated with slope gradient. (**c**) High soil erosion risk class integrated with land use. (**d**) Very high soil erosion risk class integrated with land use.

Areas with very high and high soil erosion risk are recognized as the priority areas for implementing soil and water conservation measures or for enhancing the existing management. Intersecting the areas with high and very high soil erosion risk with the slope gradient data (Figure 12a,b) revealed that approximately 84.81% and 12.37% of the very high soil erosion risk areas are located at 15°–25° and 25°–35° slopes, respectively. In addition, a total of 64.57%, 19.33% and 15.02% of high soil erosion risk areas are located at 15°–25°, 8°–15° and 25°–35° slopes, respectively. Intersecting the areas with high and very high soil erosion risk with the land use data (Figure 12c,d) revealed that about 99.57% of the very high soil erosion risk areas and 25.25% of the high soil erosion risk areas are farmland areas, and 54.60% and 19.97% of the high soil erosion risk areas are forest and grassland areas.

In total, 85.17% and 12.35% of farmland with a very high erosion risk is located at 15°–25° and 25°–35° slopes, respectively. In addition, 70.60% of forest land with a high erosion risk is located in areas with annual precipitation of more than 550 mm. These results indicate that slope and precipitation have a large influence on the erosion risk. Farmland located at 15°–35° and forest with annual precipitation larger than 550 mm are the most prior areas to implement conservation planning and management.

After identifying regions with very high and high erosion risks, appropriate measures or management should be implemented in these areas in order to control soil erosion. Conservation Agriculture can be an effective approach to conserve soil and water and promote the sustainable use of farmland in the Loess Plateau [55]. According to FAO (Food and Agriculture Organization of the United Nations), Conservation Agriculture is a farming system that promotes maintenance of a permanent soil cover, minimum soil disturbance (i.e., no tillage), and diversification of plant species. It increases water and nutrient use efficiency, improves crop production and reduces soil erosion by increasing the surface storage capacity of rainfall and reducing flow velocity [56]. Previous studies have shown that conservation tillage including no tillage, reduced tillage, mulch tillage have been implemented and achieved better effect than traditional tillage in some parts of the Loess Plateau [56,57,58]. Therefore, we suggest that Conservation Agriculture should be promoted in farmland with high erosion risk in this region. In addition, terraces can be considered as auxiliary technique for the farmland with steep slope [55]. 

Forest located in high precipitation areas should receive more attention and its monitoring should be enhanced to prevent debris flow and collapse. Moreover, water availability is the most limiting factor for vegetation growth, vegetation type and distribution, survival rate, and plant density should be considered in ecological restoration projects planning and management [55,59]. Tillage and other human intervention that will destroy soil in forest should be forbidden. 

Since open grazing without reasonable management can cause severe soil erosion in the Loess Plateau [4,60]. Forbidding open grazing on hills and grassland have been implemented in parts of Loess Plateau [61]. However, according to Cheng et al. (2014) [62], long-term grazing exclusion has a negative impact on species generation and ecosystem stability in the semi-arid region of Loess Plateau. Keeping the balance between growth and harvest is important to avoid degradation and natural resources management. They suggested that recovery grassland can be used for mowing once every two years and light grazing (two sheep/ha). Therefore, proper grazing should be carried out in grassland with high erosion risk. Captive breeding and mowing should be promoted instead of open grazing. In the same time, the government should plant grass and mix grasses with shrubs.

### 3.5. Advantages and Limitations of the Proposed Methodology

The methodology proposed in the present study illustrates the possibility of the application of the MCDA method for the evaluation of soil erosion risk by experts. This methodology overcomes one of the shortcomings of quantitative methods (such as USLE/RUSLE models), which assume that the soil erosion factors are independent, and ignore the synergic and redundant effects among these factors [63,64]. Moreover, Yuan et al. (2019) [65] reported that the soil erosion effect of certain combinations of erosion driving factors is more severe than the sum of their individual effect in the hilly-gully region. Using the MCDA methodology on the basis of non-additive measures, expert judgment for the interdependences between the soil erosion factors are considered in the processes of soil erosion risk assessment. To elaborate the advantages of MCDA methodology, a detailed comparison between the MCDA methodology and a widely applied traditional erosion model, USLE model, is made and shown in Table 5.

The purpose of the present study was to provide a flexible qualitative soil erosion risk assessment method that can help local decision makers identify areas with high erosion risk for soil and water conservation planning and management. Some previous studies have reported that the simple estimation approach using experts’ knowledge is a good approach and provides a more accurate estimation than complex models when data are limited or for large-scale implementation [19,66,67]. However, the qualitative assessment of soil erosion risk cannot avoid variations between the judgement of different experts [20,68]. Therefore, in the current study, the weights of factors were determined by experts in collaboration with local managers to obtain a more practical estimation of the erosion risk.

Because of the different influencing factors of different soil erosion types, the existing soil erosion assessment models can only be used to evaluate specific soil erosion types. Thus, another advantage of the methodology proposed in the current study is that the parameters involved in the erosion risk assessment, and their normalization and classification functions can be modified by experts according to site-specific conditions. Hence, the proposed methodology can be applied not only to evaluate water erosion, but also to evaluate wind erosion or freeze-thaw erosion, etc. Therefore, the methodology proposed in the present study can meet the comprehensive requirement for decision makers to carry out soil and water conservation measures in the future.

Although the effectiveness of the soil erosion risk assessment tool has been proposed in this study, there remain several key points to point out in the application of this method. Firstly, attributes needed for soil erosion risk assessment are identified by experts, and thus the expert must be very familiar with specific local conditions to ensure the accuracy of the results. Secondly, due to the uncertainty and errors originating from data quality, the spatial resolution of input data will influence the accuracy of the assessment results. What is more, a small number of factors should be selected for inclusion in the questionnaire for soil erosion risk assessment to avoid confusing the experts.

In summary, the presented MCDA methodology can be utilized in multiple-scale soil erosion risk assessment and can be applied to estimate multiple soil erosion types, such as water erosion, wind erosion and freeze-thaw erosion, etc. Moreover, as a flexible qualitative soil erosion risk assessment tool, the method can be modified to be employed in data limited areas.

## 4. Conclusions

In the present study, a flexible qualitative soil erosion risk assessment tool was developed using the multi-criteria decision analysis (MCDA) framework based on the Choquet integral. This method is proposed to help decision makers identify the priority remediation areas in soil and water conservation planning and management. The main innovation of this study is the soil erosion risk assessment method and indicator. Different from the traditional quantitative soil erosion models, the MCDA method considers the synergistic and inhibitory effects among soil erosion parameters. Moreover, the methodology presented in the current study combining objective data with subjective assessment based on Choquet integral, could solve the weight problem encountered when using the quantitative method. In addition, due to the important influence on soil erosion, slope aspect was investigated and considered as a main parameter in the study. Finally, the parameters identified for inclusion in the soil erosion risk assessment can be modified by experts according to the erosion types, data availability and other local conditions. A case study of the Xinshui River watershed located in the Loess Plateau of China was then presented herein to demonstrate the effectiveness of the proposed methodology. The very high and high integrated soil erosion risks areas are mostly concentrated in farmland located at 15°–35° slopes. The results are in general agreement with the judgement of local experts and managers. The case study results demonstrated that the methodology proposed in this study is an effective tool and has a possibility of providing a quick and accurate identification of high erosion risk in regions with different erosion types. Since the temporal variation of soil erosion risk maybe important and required for stakeholders and decision makers in soil and water conservation planning and management, further research will focus on the temporal variability analysis of soil erosion risk in the study area. Moreover, the method will be applied to other areas with different soil erosion types to test and verify its applicability in the future.

## Figures and Tables

**Table 1:** Data sources and formats.

Data Types	Time	Spatial Resolution	Format	Data Sources
Precipitation	year	1000 m	Raster	China Meteorological Data Service Center and Hydrological Data of Yellow River Basin of Annual Hydrological Report P. R. China [28]
DEM	2003	30 m	Raster	Geospatial Data Cloud Site, Computer Network Information Center, Chinese Academy of Sciences [29]
Land use	2015	-	Polygon	-
Soil texture	1990s	1000 m	Raster	Data Center for Resources and Environmental Sciences, Chinese Academy of Sciences (RESDC) [30]
NDVI (MOD13Q1)	2015	250 m	Raster	Land Processes Distributed Active Archive Center [27]
Socio-economic data	2015	-	Text	Shanxi statistical year book [31]

**Table 2:** Classification and normalization score of soil erosion risk attributes.

No.	Parameters	Classification	Normalization Score
1	Land use	Farmland	50
		Forest	10
		Grassland	20
		Water	0
		Residential land	0
2	Slope gradient (angle gradient)	0–5	0
		5–8	45
		8–15	80
		15–25	90
		25–35	95
		>35	100
3	Aspect	Flat	0
		Sunny slope	90
		Semi-shady and semi-sunny slope	80
		Shady slope	70
4	Precipitation (mm)	<500	75
		500–550	80
		550–600	90
		600–650	100
5	NDVI	−1–0	0
		0–0.2	100
		0.2–0.4	75
		0.4–0.6	50
		0.6–0.8	25
		0.8–1	5
6	Soil texture	loam	80
		clay loam	70

**Table 3:** Description of different land use classes.

Land Use	Description
Farmland	Dry land for planting corn and other crops with tillage
Forest	Secondary forest (*Quercus wutaishanica*), artificial forests (*Pinus tabulaeformis*, *Platycladus orientalis*, and *Robinia pseudoacacia*), Orchards and shrub
Grassland	Natural cover grassland without management
Water	Lakes, rivers and reservoirs
Residential land	Rural/urban residential sites, roads, and built-up land for industrial and mining enterprises
Unutilized land	Unutilized tillage and abandoned fields

**Table 4:** Questionnaire for experts to define the measure scores related to each possible coalition of criteria used in soil erosion risk assessment.

Slope Gradient	Precipitation	NDVI	Land Use	Soil Texture	Aspect	Score
0	0	0	0	0	0	0
1	0	0	0	0	0	40
0	1	0	0	0	0	30
0	0	1	0	0	0	25
0	0	0	1	0	0	35
0	0	0	0	1	0	20
0	0	0	0	0	1	10
1	1	0	0	0	0	75
1	0	1	0	0	0	70
1	0	0	1	0	0	80
1	0	0	0	1	0	65
1	0	0	0	0	1	60
0	1	1	0	0	0	60
0	1	0	1	0	0	70
0	1	0	0	1	0	55
0	1	0	0	0	1	45
0	0	1	1	0	0	55
0	0	1	0	1	0	50
0	0	1	0	0	1	40
0	0	0	1	1	0	60
0	0	0	1	0	1	50
0	0	0	0	1	1	35
1	1	1	0	0	0	75
1	1	0	1	0	0	85
1	1	0	0	1	0	75
1	1	0	0	0	1	75
1	0	1	1	0	0	80
1	0	1	0	1	0	75
1	0	1	0	0	1	75
1	0	0	1	1	0	80
1	0	0	1	0	1	80
1	0	0	0	1	1	75
0	1	1	1	0	0	70
0	1	1	0	1	0	65
0	1	1	0	0	1	65
0	1	0	1	1	0	70
0	1	0	1	0	1	70
0	1	0	0	1	1	65
0	0	1	1	1	0	70
0	0	1	1	0	1	70
0	0	1	0	1	1	60
0	0	0	1	1	1	70
1	1	1	1	0	0	90
1	1	1	0	1	0	80
1	1	1	0	0	1	80
1	1	0	1	1	0	90
1	1	0	1	0	1	90
1	1	0	0	1	1	80
1	0	1	1	1	0	85
1	0	1	1	0	1	85
1	0	1	0	1	1	75
1	0	0	1	1	1	85
0	1	1	1	1	0	70
0	1	1	1	0	1	70
0	1	1	0	1	1	65
0	1	0	1	1	1	70
0	0	1	1	1	1	65
1	1	1	1	1	0	95
1	1	1	1	0	1	90
1	1	1	0	1	1	75
1	1	0	1	1	1	85
1	0	1	1	1	1	80
0	1	1	1	1	1	70
1	1	1	1	1	1	100

**Table 5:** Comparison between the MCDA methodology and USLE model.

Items	MCDA Methodology	USLE Model [63,64]
Input	Rainfall, slope, aspect, land use, soil texture and NDVI	Rainfall-runoff erosivity factor, soil erodibility factor, slope length and the slope steepness factor, land cover and management factor and the support and conservation practices factor
Output	Soil erosion risk score and class	Average annual soil loss
Erosion types	Multiple erosion types	Water erosion
Assessment scale	Multiple scale	Large scale
The synergistic and inhibitory effects and different importance among erosion factors	Considered by expert judgement	Not considered
Advantages and disadvantages	Commend in Section 3.5	Widely applied No consideration of the synergistic and inhibitory effects and different importance among erosion factors
